# A Case of Epicardial Ablation of the Sinus Node for the Treatment of Inappropriate Sinus Tachycardia

**DOI:** 10.19102/icrm.2025.16093

**Published:** 2025-09-15

**Authors:** Mahmoud Eldesouky, Noha Elbanhawy, Shajil Chalil, Khalid Abozguia

**Affiliations:** 1 Cardiology Department, Blackpool Teaching Hospital NHS Trust, Blackpool, UK; 2 Cardiology Department, Marshall University, Joan C. Edwards School of Medicine, Huntington, WV, USA

**Keywords:** Case report, hybrid ablation, inappropriate sinus tachycardia, surgical window

## Abstract

Despite advancements in medical therapy, managing symptomatic inappropriate sinus tachycardia (IST) remains challenging. The role of catheter ablation in addressing this condition remains ambiguous according to multiple cardiac society guidelines. In this case study, we illustrate the efficacy of a hybrid approach involving sinus node modification and ablation in a patient with refractory symptoms, while also addressing the associated challenges and safety considerations of this procedure. A 58-year-old female patient was troubled with recurrent palpitations secondary to IST. Due to the proximity of the target ablation site to the phrenic nerve, this area was not amenable to complete ablation endocardially. To alleviate symptoms, an ablation procedure was planned, aiming for epicardial sinus node modification and displacement of the phrenic nerve from the target site. The procedure was completed under general anesthesia. The conventional subxiphoid technique was deemed challenging even with a surgical approach due to the patient’s body habitus and significantly increased body mass index; hence, she underwent a 5-cm right anterior thoracotomy to establish access to the pericardium. The sinoatrial (SA) node was ablated surgically by direct application under vision of the right atrium around the area of the SA node to avoid the phrenic nerve. Modification and ablation of the sinus node in patients exhibiting features of IST may be considered to help alleviate patients’ symptoms. Further follow-up and assessments with large cohorts and powered randomized controlled studies are needed. Our case represents an example where a hybrid invasive approach resulted in a safe procedure with immediate symptomatic benefit.

## Introduction

Despite medical therapy advancements, managing symptomatic inappropriate sinus tachycardia (IST) remains challenging. The role of catheter ablation remains ambiguous according to multiple guidelines.^1,2^ We illustrate the efficacy of a hybrid approach to sinus node modification, addressing the procedure’s challenges and safety considerations.

## Case presentation

A 58-year-old female patient was troubled by recurrent palpitations secondary to IST despite multiple rate-control medications at maximum tolerated doses (10 mg of bisoprolol twice a day, 125 μg of digoxin once a day, and 7.5 mg of ivabradine twice a day). The patient had ischemic cardiomyopathy, and a biventricular (BiV) implantable cardioverter-defibrillator was implanted for primary prevention given the persistent severe left ventricular systolic dysfunction and left bundle branch block despite guideline-directed medical therapy. Her past medical history revealed hypothyroidism, type 2 diabetes mellitus, mixed hyperlipidemia, essential hypertension, and a high body mass index (>40 kg/m^2^).

The patient underwent an extensive evaluation and was discussed in multiple multidisciplinary team meetings. All potential triggers, including obstructive sleep apnea, anemia, and ischemia, were excluded. Management of her comorbidities was optimized medically.

She remained symptomatic with palpitations and New York Heart Association (NYHA) class III. The BiV pacing percentage was significantly low due to a high sinus rate. The BiV device was programmed to the VVI mode as the patient had a high percentage of tracked elevated sinus tachycardia, which was poorly tolerated. This was to help avoid tracking the sinus tachycardia by pacing only the ventricles, reducing unnecessary pacing-induced tachycardia episodes.^3^ The adjustment was necessary to prevent rapid atrial tracking resulting in hemodynamic instability and increased myocardial oxygen demand.^4,5^ The patient was thus listed for an electrophysiology (EP) study and sinus node modification with a view toward lowering the sinus rate, aiming to improve the BiV pacing percentage. Three-dimensional (3D) mapping confirmed the diagnosis of IST. The EP study ruled out an accessory pathway with evidence of dual atrioventricular (AV) node physiology and single echo.

**Figure 1:**
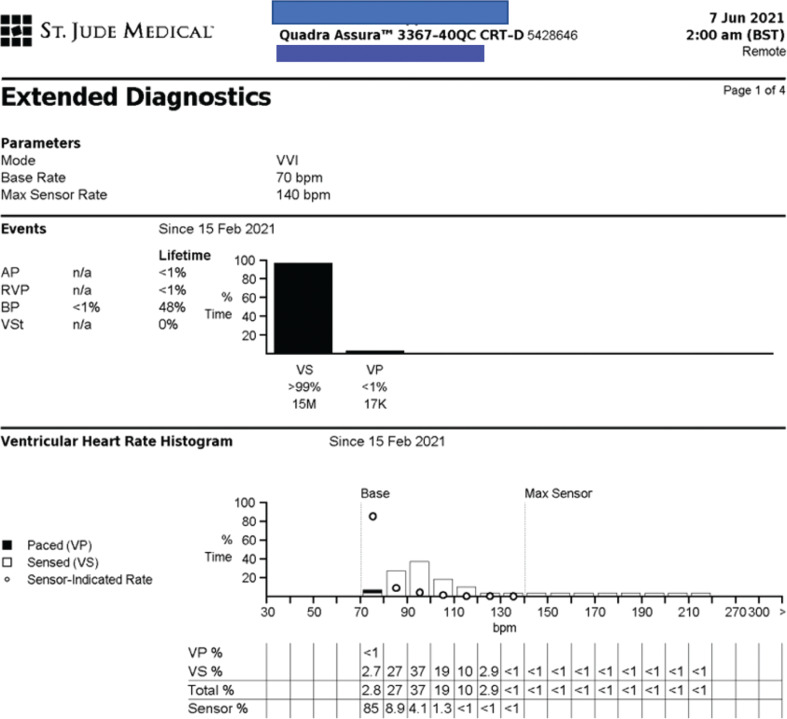
Ventricular heart rate histogram before ablation. Note the almost-complete absence of ventricular pacing with heart rates predominantly between 90 and 100 bpm.

The 3D activation mapping showed the earliest atrial signal in an area close to the sinus node. An endocardial ablation attempt was made. Due to the proximity of the target ablation site to the phrenic nerve, this area was not amenable to complete ablation endocardially. Device interrogations continued to show persistent sinus tachycardia **(Figure 1)**. The patient was highly symptomatic, and BiV pacing was inhibited because of high intrinsic rates.

To alleviate symptoms, an ablation procedure was planned, aiming for epicardial sinus node modification and displacement of the phrenic nerve from the target site. This option was more invasive, but we predicted difficulty in obtaining percutaneous pericardial access with the standard subxiphoid technique given the patient’s body habitus, and thus we arranged for the cardiothoracic surgical team to assist with a surgical window.

**Figure 2:**
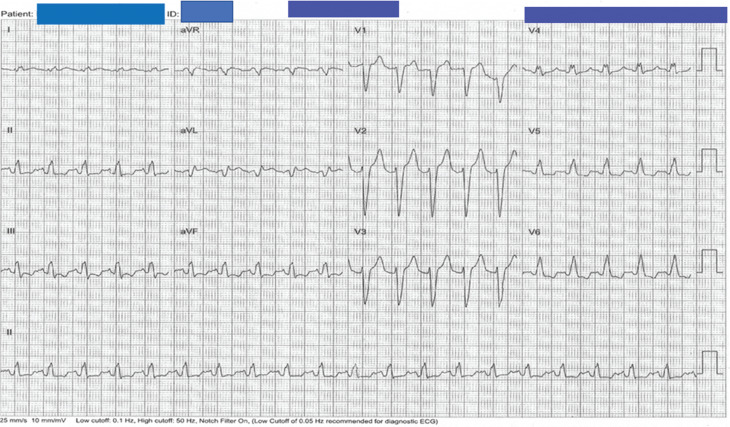
Baseline electrocardiogram taken prior to the procedure showing sinus tachycardia.

**Figure 3:**
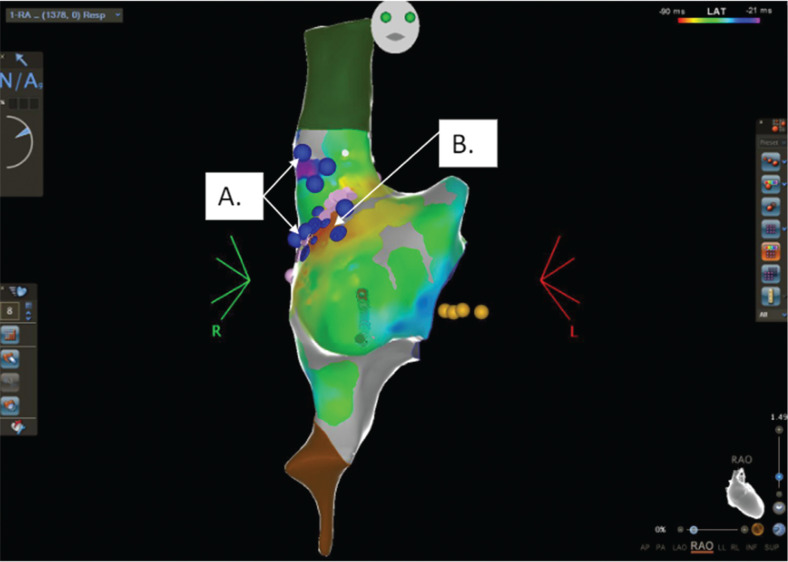
Baseline CARTO^®^ map anterior–posterior view showing the area of earliest activation in the sinus node region. The yellow dots represent the His area. **A:** Phrenic nerve tags in blue. **B:** Area of earliest activation.

The procedure was performed under general anesthesia in a hybrid EP lab equipped with 3D mapping capability and a surgical facility. Baseline electrocardiogram showed sinus tachycardia **(Figure 2)**. Ultrasound guidance was used for right femoral vein access. 3D voltage and activation maps **(Figure 3)** were created using the CARTO^®^ 3 mapping system (Biosense Webster, Inc., Diamond Bar, CA, USA) and a high-density mapping catheter (PentaRay^®^ NAV Catheter; Biosense Webster). Isoprenaline was used during mapping to identify the sites of fastest sinus activity. The earliest signal was confirmed to be in the sinus node region in an area with phrenic nerve capture, which previously precluded endocardial ablation **(Figure 3)**. The patient underwent a 5-cm right anterior thoracotomy, establishing access to the pericardium.

The SA node was ablated surgically using the cryoICE cryotherapy probe (AtriCure Inc., Mason, OH, USA) by direct application under vision of the right atrium around the area of the SA node to avoid the phrenic nerve. Multiple single-application freezes with temperatures ranging between −50°C and −70°C were achieved to guarantee irreversible tissue damage. A number of lesions were performed epicardially. Further lesions were created as directed by the EP team to achieve electrical modification and ablation of the sinus node.

**Figure 4:**
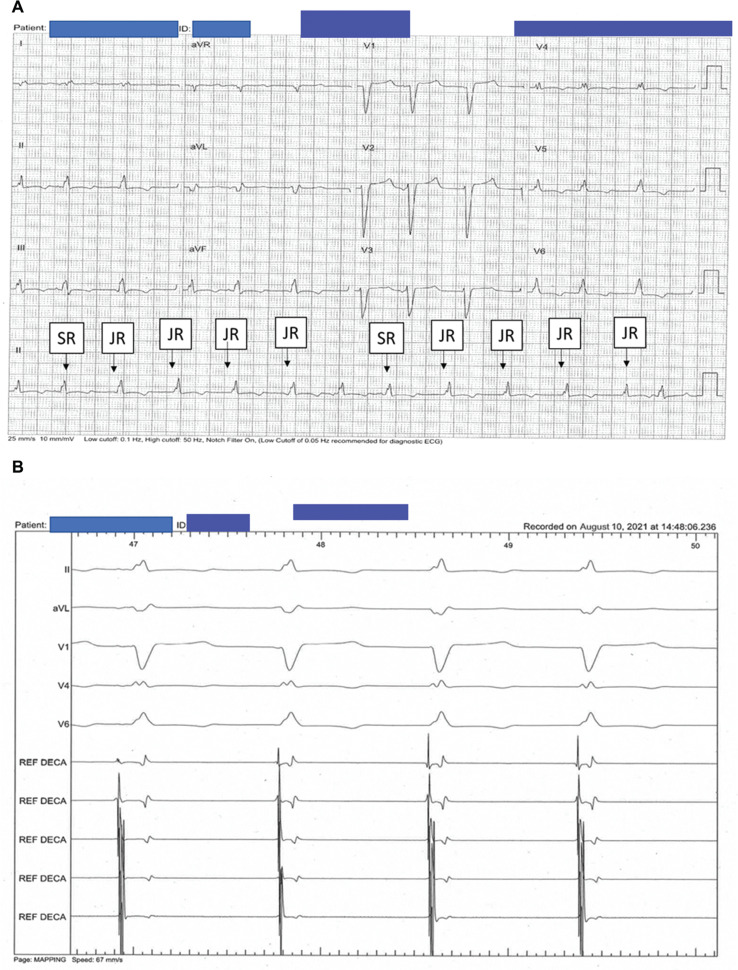
**A:** Electrocardiogram taken after sinoatrial node ablation. A transition from sinus to junctional rhythm is seen on the long strip and limb leads. **B:** Electrogram taken during ablation. Intracardiac tracing showing transition from sinus (first beat) to junctional rhythm beats. *Abbreviations:* JR, junctional rhythm; SR, sinus rhythm.

**Figure 5:**
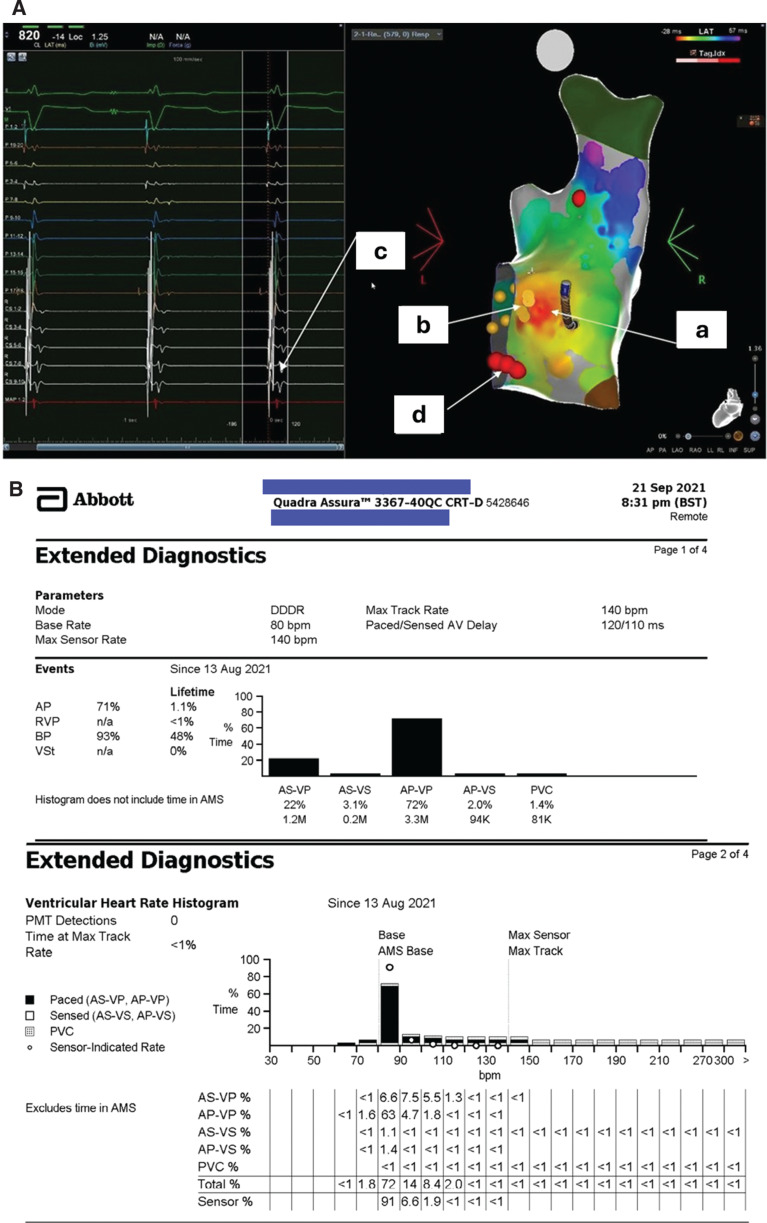
**A:** CARTO^®^ map (posteroanterior view) after epicardial sinus node ablation with earliest activation in the low right atrium. **a:** Earliest area of activation—red area of activation mapping. **b:** Yellow tags in the His position. **c:** Coronary sinus electrogram showing junctional rhythm post-ablation. **d:** Red tags for ablation lesions in the slow pathway region. **B:** Ventricular heart rate histogram post-ablation. Note predominantly paced rhythm with the resting rate at a programmed base rate of 80 bpm. Higher rates at upper tracking rate.

At this point in the procedure, the heart rate went down from 100 to 60 bpm and the rhythm changed to a junctional rhythm **(Figures 4A and 4B)**. Slow pathway modification was also performed during sinus rhythm in view of dual AV node physiology and echoes. Remapping of the right atrium confirmed the area of earliest activation to be in the His region **(Figure 5A)**. The incision was closed surgically. The device was then programmed to DDD pacing at 80 bpm with consistent BiV pacing, with plans to gradually reduce the heart rate in stages as an outpatient. This is given that gradual rate reduction allows the autonomic nervous system and cardiac conduction pathways to adjust more smoothly, thereby reducing the risk of destabilizing arrhythmic events.^6^ On follow-up, device interrogation showed a predominantly paced atrial and BiV rhythm at the programmed base rate with a physiologic increase in rates that were sensor-driven **(Figure 5B)**. After the procedure, the patient exhibited a dramatic improvement in her symptoms of palpitations with an optimal percentage of BiV pacing. A perceived improvement in heart failure symptoms to NYHA class II was also observed.

Consent for publication was obtained, in line with the Committee on Publication Ethics best practice guidelines, and the individual who is being reported on is aware of the possible consequences of that reporting.

## Discussion

The patient in this case met the criteria for the diagnosis of IST as defined in the guidelines and consensus statements.^7–9^ Her average heart rate exceeded 90 bpm as seen in the ventricular rate histogram prior to ablation, and she had distressing symptoms. With continuous monitoring, her heart rates showed fluctuation and included normal and increased resting rates at times but excessive rates with exercise; intermittent rapid unexplained rates occurred with or without positional changes.

The mechanisms of IST are either related to the dysregulation of autonomic inputs to the sinus node or intrinsic sinus node disease. Different theories exist, including the presence of a β-receptor–stimulating autoantibody, a β-receptor super-sensitivity, a muscarinic (M2) receptor abnormality, impaired baroreflex control, a blunted response to adenosine with or without autonomic blockade, a reduction in and/or alteration of baroreflex gain, and abnormalities in parasympathetic function despite the preservation of sympathovagal balance and vagal denervation.^7,10^

There is still debate regarding the clinical impact of high resting heart rates. In the Tromsø study, elevated resting heart rates were associated with an increased risk of myocardial infarction and mortality.^11^

A meta-analysis from the Asia Pacific Cohort Studies Collaboration concluded that a resting heart rate of >65 bpm had a strong independent effect on premature mortality and stroke.^12^

The patient’s debilitating symptoms despite optimal medical management prompted the more-invasive approach. Options considered were AV node ablation or modification/ablation of the sinus node. Because AV node ablation was less physiologic and unlikely to control the tachycardia, as the device would most likely exhibit upper tracking rate behavior, ablation strategies targeting the sinus node were more favored.

Surgical and catheter ablation of the sinus node are not currently recommended but may be considered in the most symptomatic patients after the failure of other therapies.^1,13^

In our case, the limitation of endocardial ablation due to potential damage to the phrenic nerve led us to consider a surgical hybrid approach. While more invasive, this method eliminates that risk by allowing direct visualization and avoidance of the phrenic nerve during ablation. Percutaneous epicardial access with phrenic nerve displacement using a balloon was also considered,^14^ but it was deemed technically challenging due to body habitus.

Pulsed field ablation (PFA) is an emerging ablation modality that is rapidly growing in popularity. One of the main advantages of PFA is its preferential tissue ablation characteristics, which allow for avoiding phrenic nerve injury.^15^ There is still a need for appropriate caution, however, as there are still only limited data available in the literature quantifying the effects of PFA on phrenic nerve stunning and their long-term impact.^16^

Our case represents an example where a hybrid invasive approach resulted in a safe procedure with immediate symptomatic benefit. Modification and ablation of the sinus node in patients exhibiting features of IST may be considered to help alleviate symptoms. Further assessment with large cohorts and powered randomized controlled studies is needed.
